# The colorectal cancer epidemic: challenges and opportunities for primary, secondary and tertiary prevention

**DOI:** 10.1038/s41416-018-0264-x

**Published:** 2018-10-04

**Authors:** Hermann Brenner, Chen Chen

**Affiliations:** 1 Division of Clinical Epidemiology and Aging Research, German Cancer Research Center (DKFZ), Heidelberg, Germany; 2 Division of Preventive Oncology, German Cancer Research Center (DKFZ) and National Center for Tumor Diseases (NCT), Heidelberg, Germany; 3 German Cancer Consortium (DKTK), German Cancer Research Center (DKFZ), Heidelberg, Germany; 4 Medical Faculty Heidelberg, University of Heidelberg, Heidelberg, Germany

## Abstract

Colorectal cancer (CRC) is both one of the most common and one of the most preventable cancers globally, with powerful but strongly missed potential for primary, secondary and tertiary prevention. CRC incidence has traditionally been the highest in affluent Western countries, but it is now increasing rapidly with economic development in many other parts of the world. CRC shares several main risk factors, such as smoking, excessive alcohol consumption, physical inactivity and being overweight, with other common diseases; therefore, primary prevention efforts to reduce these risk factors are expected to have multiple beneficial effects that extend beyond CRC prevention, and should have high public health impact. A sizeable reduction in the incidence and mortality of CRC can also be achieved by offering effective screening tests, such as faecal immunochemical tests, flexible sigmoidoscopy and colonoscopy, in organised screening programmes which have been implemented in an increasing number of countries. Countries with early and high uptake rates of effective screening have exhibited major declines in CRC incidence and mortality, in contrast to most other countries. Finally, increasing evidence shows that the prognosis and quality of life of CRC patients can be substantially improved by tertiary prevention measures, such as the administration of low-dose aspirin and the promotion of physical activity.

## Introduction

Colorectal cancer (CRC), including cancer of the colon and rectum (ICD-10 positions C18–C20), is the third most common cancer globally, with an estimated number of 1.4 million diagnoses in 2012.^1^ Incidence has traditionally been the highest in affluent Western countries, but is now rapidly increasing with economic development in many other parts of the world. The incidence and mortality of CRC strongly increases with age, and the median age of diagnosis is close to 70 years in developed countries. Aside from age, well-established risk factors include male sex, smoking, excessive alcohol consumption, physical inactivity, high consumption of red and processed meat, being overweight and having a family history of CRC. For the vast majority of cases, initial treatment includes surgery, with minimally invasive surgery being increasingly offered.^2^ Surgical therapy is commonly supplemented by neoadjuvant radiotherapy for stage-II and stage-III rectal cancer, and by adjuvant chemotherapy for high-risk stage-II and stage-III colon cancer.^3–5^ Five-year relative survival has steadily increased over recent decades and now exceeds 65% in the most affluent countries, including the United States.^6^ However, much lower survival rates are still observed in many parts of the world, including several European countries.^7^ By far, the strongest prognostic factor is stage at diagnosis, with 5-year relative survival rates ranging from ∼90% for patients who are diagnosed in the localised stage, to just over 10% for patients diagnosed with distant tumour spread, emphasising the importance of early detection.^8^

In this article, we provide an overview of recent and expected future trends in the incidence and mortality of CRC, demonstrating the challenges of the CRC epidemic that is ongoing in most parts of the world. This overview will be followed by summaries of current strategies and opportunities to cope with this epidemic by primary, secondary or tertiary prevention. We will conclude our review by deriving recommendations for the implementation of preventive strategies and areas to focus on in future research.

### The CRC epidemic

**Fig. 1:**
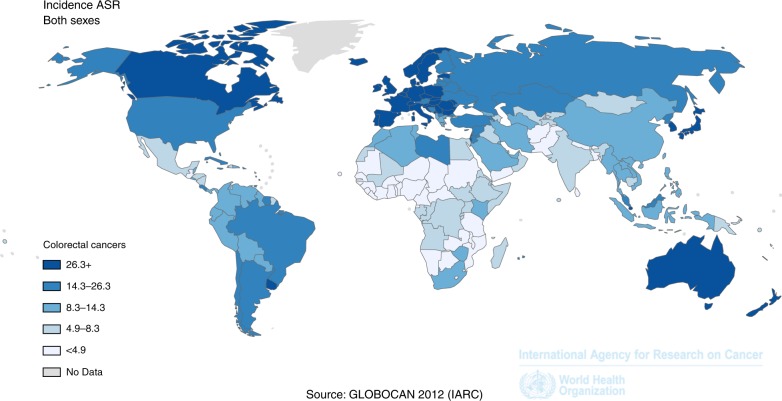
Estimated age-standardised incidence rate (standard: world population) of colorectal cancer in 2012 (source: Globocan 2012, International Agency for Research on Cancer^1^)

Incidence rates of CRC vary by up to tenfold across countries worldwide, with a distinct positive gradient according to economic development. The highest levels of age-standardised CRC incidence are observed in the most affluent countries, such as Australia and some European countries (around 40 per 100,000 for both sexes combined in 2012) (Fig. 1).^1,9^ Age-standardised incidence rates are ∼50% higher among men than among women in most countries. While incidence has stabilised at high levels or even started to decline in a few highly developed countries, incidence rates continue to increase strongly alongside economic development in most low- and middle-income countries. Substantially stronger increases in global crude incidence and numbers of cases are expected in the decades to come, owing to population growth and demographic ageing. Demographic changes alone are expected to lead to an increase in the number of new CRC cases by 79% worldwide, from ∼1.4 million in 2012 to > 2.4 million in 2035.^1^

**Fig. 2:**
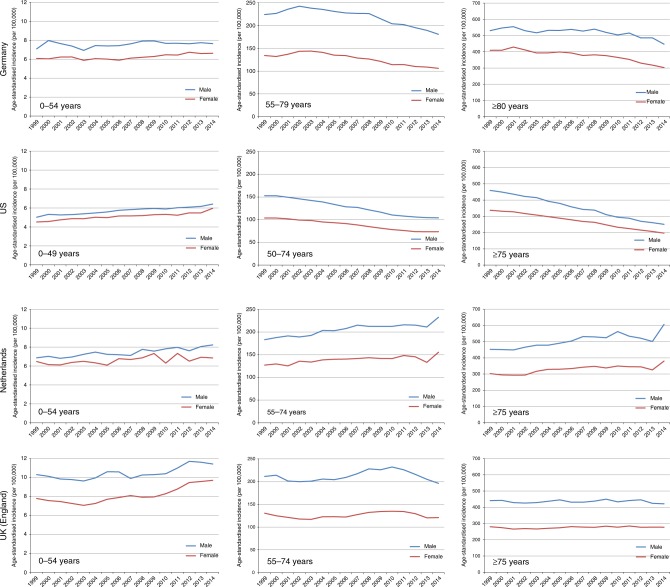
Trends in age-standardised CRC incidence (standard: world population) among pre-screening age groups, screening-eligible groups and the very elderly in affluent countries with long-standing (Germany and the United States) or recent (Netherlands and the United Kingdom) CRC screening programs, 1999–2014 (data sources:^100–103^)

Overall incidence and mortality rates appear to have stabilised at high levels, or even started to decline in a few affluent countries in which effective CRC screening has become widespread, such as the United States and Germany. However, an opposing pattern is now being observed in younger generations, with increasing incidence rates in birth cohorts and populations who have not yet reached screening age. These increases are also observed in those more affluent countries, pointing to the unfavourable trends in important CRC risk factors in younger generations (Fig. 2).^8–12^

## Modifiable factors associated with CRC: opportunities for primary prevention

Besides some non-modifiable risks and protective factors such as sex, age, family history and genetic predisposition, epidemiological studies have revealed a number of potentially modifiable factors that are associated with an increased or decreased risk of CRC. These point to challenges and potential opportunities for primary prevention. Established risk factors include cigarette smoking,^13^ excessive alcohol consumption,^14^ being overweight or obese^15^ and consuming high amounts of red and processed meat.^16^ On the other hand, physical activity,^17^ regular use of aspirin^18,19^ and hormone replacement therapy (HRT)^20^ have been found to be associated with a reduced CRC risk; there are indications that consumption of milk and whole grains might also confer a protective role against CRC.^16^ Table 1 provides an overview on the results of recent meta-analyses.

**Table 1:** Relative risks for colorectal cancer-modifiable risk factors and protective factors according to recent meta-analyses

Factor	Reference	No. of studies	No. of cases	Indicators of risk or protective factor	Pooled relative risk (95% CI)
*Risk factor*					
Consumption of red and processed meat	WCRF CUP^99^	8	6662	Red meat, per 100 g/day	1.12 (1.00−1.25)
		10	10,738	Processed meat, per 50 g/day	1.16 (1.08−1.26)
Alcohol consumption	WCRF CUP^99^	16	15,896	Per 10 g/day	1.07 (1.05–1.08)
Body fatness	WCRF CUP^99^	38	71,089	BMI, per 5 kg/m^2^	1.05 (1.03–1.07)
		8	4301	Waist circumference, per 10 cm	1.02 (1.01–1.03)
		4	2564	Waist:hip ratio, per 0.1 unit	1.02 (1.01–1.04)
Smoking	Botteri et al.^13^	106	39,779	Ever vs never smokers	1.18 (1.11–1.25)
				Current vs never smokers	1.07 (0.99–1.16)
				Former vs never smokers	1.17 (1.11–1.22)
*Protective factor*					
Physical activity^a^	WCRF CUP^99^	12	8396	Total physical activity, highest vs lowest levels	0.80 (0.72–0.88)
		20	10,258	Recreational physical activity, highest vs lowest levels	0.84 (0.78–0.91)
Consumption of whole grains	WCRF CUP^99^	6	8320	Per 90 g/day	0.83 (0.78−0.89)
Consumption of food containing dietary fibre	WCRF CUP^99^	21	16,562	Per 10 g/day	0.93 (0.87−1.00)
Consumption of dairy products	WCRF CUP^99^	10	14,859	Dairy products, per 400 g/day	0.87 (0.83−0.90)
		9	10,738	Milk, per 200 g/day	0.94 (0.92–0.96)
		7	6462	Cheese, per 50 g/day	0.94 (0.87–1.02)
		10	11,519	Dietary calcium, per 200 mg/day	0.94 (0.93–0.96)
Aspirin	Algra et al.^18^	26^b^	25,618	Any aspirin vs non-user	0.67 (0.60–0.74)
		17^b^	12,659	Maximum reported aspirin vs non-user	0.62 (0.58–0.67)
Hormone replacement therapy	Green et al.^20^	30	6256^c^	Any hormone replacement, ever vs never use	0.84 (0.81–0.88)
		16	2285^c^	Oestrogen-only hormone replacement, ever vs never use	0.83 (0.79–0.86)
		17	1355^c^	Oestrogen + progestogen hormone replacement, ever vs never use	0.81 (0.75–0.87)

*CI* confidence interval, *WCRF CUP* World Cancer Research Fund continuous update project

^a^For colon cancer only

^b^Major results are based on 26 and 17 case–control studies

^c^Number of cancer cases exposed to hormone replacement use

### Risk factors for CRC

Although the overall prevalence of tobacco smoking among men has decreased globally by ∼10% from 1980 to 2013, nearly one out of three men currently smokes.^21^ However, trends have been very diverse across countries, with stronger reductions in smoking prevalence seen in countries implementing effective tobacco control policies, compared with very modest or no reductions, or even further increases, in smoking prevalence in countries with limited or no effective tobacco control policies. Similarly, diverse trends in smoking prevalence have been observed among women, although substantially fewer women than men smoke in most countries. Comprehensive implementation of effective tobacco control policies, along with support for smokers to quit smoking, has the potential to substantially reduce the CRC epidemic, in addition to reducing the burden of other smoking-related cancers and common smoking-related diseases, such as cardiovascular diseases (CVD).

Overweightness, obesity and their associated adverse metabolic consequences, starting from childhood and adolescence, have reached epidemic levels globally,^22^ and might be primary drivers of the increases in CRC incidence at younger ages even in countries where the overall CRC incidence has started to decline.^23,24^ Cutting the obesity epidemic by primary prevention efforts, especially among young families, will therefore be crucial in the coming decades for coping not only with the CRC epidemic, but also epidemics of other obesity-related chronic diseases, such as many other cancers and diabetes.

While the relationship with CRC risk of these and other modifiable risk factors such as high consumption of alcohol, red and processed meat are well established, ongoing research focuses on the variation of these relationships according to molecular CRC subtypes. The emerging field of ‘molecular pathological epidemiology’ has the potential to provide enhanced insights into the underlying molecular mechanisms, which may provide enhanced approaches to primary prevention in the future.^25–29^ For example, with an ∼60% increase in risk for ever smokers, the association of smoking was found to be much stronger with tumours characterised by high microsatellite instability (MSI-H cancers) than with other molecular subtypes of CRC for which the increase was only around 10%.^30^ The majority of MSI-H cancers occur due to methylation-induced silencing of the *MLH1* gene, pointing to a potential role of smoking in (potentially reversible) DNA methylation changes.

### Protective factors for CRC

Primary prevention efforts should include the promotion of physical activity, a major preventive factor for obesity^31^ and by itself a major preventive factor for CRC, as well as encouraging healthy dietary habits with limited red and processed meat intake and adequate intake of whole grains, fibre and dairy products.

Regular use of aspirin has long been recommended for the secondary prevention of CVD. However, in 2016, the U.S. Preventive Services Task Force (USPSTF) also recommended the use of low-dose aspirin for the primary prevention of CVD and CRC in adults aged 50–59 years who have a 10% or greater 10-year CVD risk, are not at increased risk for bleeding, have a life expectancy of at least 10 years, and are willing to take low-dose aspirin daily for at least 10 years.^32^ Possible mechanisms of chemoprevention of CRC by aspirin include inhibition of the cyclooxygenase (COX) pathway or COX-independent mechanisms, such as the PIK3CA pathway, or therapy-induced senescence of cancer cells.^33^ In addition, the USPSTF recommended that the decision to initiate low-dose aspirin use for the primary prevention of CVD and CRC in adults aged from 60 to 69 years who have a 10% or greater 10-year CVD risk should be an individual one, dependent on additional factors such as individual life expectancy or risk of bleeding. The USPSTF rated current evidence to be insufficient to assess the balance of benefits and harms of initiating aspirin use for the primary prevention of CVD and CRC in adults below 50 or above 70 years of age. Thus, recommendations on the potential use of low-dose aspirin for the primary prevention of CRC should be viewed in the context of the pleiotropic effects of aspirin, including its beneficial effects on CVD and its potential adverse effects, especially with respect to gastrointestinal bleeding.

In contrast to a potential role for low-dose aspirin for primary CRC prevention, there is no such role for HRT, despite its established inverse association with CRC risk.^20^ This is because the detrimental effects of HRT on other health outcomes, such as CVD, venous thromboembolic disease and breast cancer, might well exceed the potential benefits with respect to CRC prevention.^34,35^

## Effective screening methods: opportunities for secondary prevention

In contrast to other cancers, in most cases CRC develops very slowly over many years, if not decades, following the initial transformation of a normal colorectal epithelium to an adenoma.^36^ The slow progression through the adenoma–carcinoma sequence, with the possibility of detecting and removing adenomas at colonoscopy, offers great opportunities for the secondary prevention of CRCs, in addition to the opportunities for secondary prevention of deaths from CRC by detecting the cancer at an earlier, often-curable stage.

Established screening options for CRC include endoscopic examinations of the large bowel (particularly flexible sigmoidoscopy and colonoscopy) and stool tests (e.g. faecal occult blood tests [FOBTs]). Other screening options such as capsule endoscopy or computed tomography (CT), and other stool-based tests such as DNA-based tests and blood or urine tests, are so far not competitive, either in terms of diagnostic performance or cost-effectiveness, or in the case of CT, owing to their side effects. However, an ongoing worldwide extensive search for novel biomarkers, such as blood-based ‘omics signatures’, is expected to substantially broaden and potentially enhance the portfolio of non-invasive or minimally invasive CRC screening tests.

### Stool testing

The efficacy of annual or biennial testing for CRC by FOBT, with colonoscopic follow-up of positive test results and removal of precancerous lesions, in reducing CRC incidence and mortality, has long been established by several randomised controlled trials (RCTs), such as the Nottingham trial from the United Kingdom and the Minnesota trial from the United States.^37–39^ After several decades of follow-up, reductions in CRC mortality by up to 20–30% have been observed. The type of FOBT that was available several decades ago at the beginning of these trials was guaiac-based (gFOBT), but since then, newer immunological FOBTs, commonly called iFOBTs or faecal immunochemical tests (FITs), have been developed and these offer a variety of advantages over gFOBTs. They are specific to human haemoglobin and therefore do not require dietary restrictions. Furthermore, only one stool sample from a single bowel movement (rather than three samples from three consecutive bowel movements) is required, leading to higher adherence rates in population-wide screening.^40^ Most importantly, FITs have been shown to have substantially higher sensitivity, not only for detecting CRC (typically around 60–80% compared with 30–40% for gFOBTs), but also for detecting advanced adenomas (typically around 20–30% vs ∼10%).^41–43^ FIT-based screening may therefore lead to even greater reductions in CRC mortality when compared with gFOBT-based screening, and FITs are now widely recommended and offered for CRC screening in an increasing number of countries.^44,45^ When offered in the context of organised screening programmes, with pre-announcement letters, personal invitation letters that include the test kits, and reminder letters, FIT-based screening can achieve high adherence rates of > 60% for single screening rounds and > 70% over several biennial rounds of screening.^46^

Even higher sensitivity can be achieved with a multitarget stool DNA test that combines testing for human haemoglobin and specific tumour-related DNA markers in stool,^47^ but the specificity of the test is lower than that of FITs, whereas the overall diagnostic performance remains similar.^48,49^ In addition, the logistics of stool collection and shipping are much more complex (an entire bowel movement is needed for the test) and costs are ∼20-fold higher, compared with FITs.^50^ Although this test is broadly offered in the United States following FDA approval in 2014, it is not widely recommended, offered or used in other countries.

### Endoscopy screening

Multiple RCTs conducted in the United Kingdom, Italy, Norway and the United States have consistently demonstrated the benefit of endoscopy, through a substantial reduction in tumour incidence (and associated mortality) in the distal colon and rectum (but not in the proximal colon) by once-only flexible sigmoidoscopy.^51–54^ In a meta-analysis of the results available by 2014, the relative risk (95% confidence interval) of distal CRC incidence and mortality was estimated to be 0.69 (0.63–0.74) and 0.54 (0.43–0.67) in intention-to-screen analysis and 0.58 (0.47–0.71) and 0.39 (0.21–0.73) in per-protocol analysis, respectively.^55^ Longer-term follow-up data subsequently published from two of the trials have corroborated these findings^51,53^ and demonstrated a persisting strong protection from distal CRC incidence and mortality throughout a follow-up time of up to 17 years.^51^

Long-term screening colonoscopy results from RCTs will not become available before the mid-2020s.^56^ However, evidence from multiple observational studies suggests that, compared with flexible sigmoidoscopy, colonoscopy is associated with an even stronger reduction in the incidence of CRC and mortality from cancer in the distal colon and rectum, with additional, albeit somewhat less pronounced, protection from cancer in the proximal colon. Meta-analyses of epidemiological studies published up until 2014 yielded estimates of relative risk of 0.31 (0.12–0.77) and 0.32 (0.23–0.43) for overall CRC incidence and mortality, respectively,^55^ and these findings have also been further corroborated by more recently published results.^57^ The reported relative risk estimates from both RCTs and observational studies will most likely underestimate the true effects of screening endoscopy, owing to contamination of the comparison groups by diagnostic colonoscopies, which are expected to provide similar protection from CRC through detection and removal of adenomas.^58^

Intriguingly, in the United States, which is the country with the highest reported colonoscopy uptake rate among older adults in the world,^11^ CRC incidence and mortality above the age of 50 (the previously recommended starting age for screening colonoscopy in the average risk population) have declined by approximately one-third since the beginning of this century.^8^ Similar trends among older adults have also been observed in Germany (Fig. 2),^10^ where screening colonoscopy was introduced in 2002 and diagnostic colonoscopies are likewise commonly employed.^11^ According to recent estimates, a further reduction by another 30–40% should be possible by more complete adherence to screening offers.^59^ By contrast, CRC incidence and mortality continue to increase in younger age groups not covered by screening.^10^ No decline in the incidence and mortality of CRC, or potentially even further increases, is seen in many other affluent countries in which effective screening programmes have not been initiated.^9^ In response to the increasing CRC incidence below the age of 50, the American Cancer Society recently reduced the recommended age for initiating CRC screening in the average risk population from 50 to 45 years.^60^

### Advances in risk-adapted screening

An important aspect of CRC screening is the potential to tailor screening offers depending on individual CRC risk. A positive family history has so far been the only factor recommended for such risk stratification. First-degree relatives of CRC patients are advised to start CRC screening at a younger age than the average risk population, this may be advised from 40 years of age, or at least 10 years before the youngest age at diagnosis of an affected first-degree relative.^44^ Recent evidence suggests that such risk stratification could be substantially enhanced in the future through the use of polygenetic risk scores based on emerging results from genome-wide association studies.^61–64^

### Modelling for evaluation and timely optimisation of screening programmes

Although RCTs are commonly thought to provide the highest possible evidence for the efficacy of specific screening offers under ideal (i.e. trial) conditions, the very slow development of most CRCs through the adenoma–carcinoma sequence implies that such RCTs require decades of follow-up before the final results are obtained. Consequently, the screening options initially offered might be considerably outdated by the time the results become available, and contamination of the results by the interim spread of screening and diagnostic technologies that are expected to emerge during follow-up is of concern. Furthermore, the very large sample sizes needed for RCTs typically prohibit conducting these trials on a broad variety of potential design options of screening offers, such as age at screening initiation, time intervals between screening tests, combinations of screening tests offered, etc. Alternative options for generating evidence are therefore needed, in order to make informed timely decisions in designing and steadily improving screening offers, and planning resource allocation according to the expected long-term outcomes. Markov models and microsimulation models based on the natural history of the disease can be very useful tools for modelling effectiveness and cost-effectiveness of alternative screening options. They have been successfully employed in a few countries,^65–69^ but there is much room for further development and application for enabling timely optimisation of screening programmes.

## Factors enhancing prognosis: opportunities for tertiary prevention

Increasing evidence from epidemiological studies indicates that several factors with a major impact on the risk of developing CRC are also related to the survival of CRC patients, which highlights opportunities for tertiary prevention (Table 2). Although changes in unhealthy lifestyle factors are generally difficult to achieve, the chances of accomplishing them might be much greater after the ‘teachable moment’ of a cancer diagnosis.

**Table 2:** Summary of modifiable factors that are associated with CRC risk and prognosis

Factor	Effect on CRC risk	Reference	Effect on CRC survival	Reference
Smoking	↑	Botteri et al.^13^	↓	Walter et al.^70^, Ordonez-Mena et al.^72^
Heavy alcohol consumption	↑	WCRF CUP^99^	↓	Walter et al.^73^
Overweightness and obesity	↑	WCRF CUP^99^	Inconsistent results	Lee et al.^84^, Walter et al.^85^
Vitamin D deficiency	↑	Garland et al.^94^	↓	Zgaga et al.^95^, Maalmi et al.^96^
Physical activity	↓	WCRF CUP^99^	↑	Van Blarigan et al.^80^, Otto et al.^81^
Aspirin	↓	Algra et al.^18^	↑	Li et al.^89^

*CRC* colorectal cancer, *WCRF CUP* World Cancer Research Fund continuous update project

### Associations of modifiable risk factors for CRC with patient survival

Smoking and heavy alcohol consumption, the major risk factors for CRC, also seem to be associated with lower survival rates in CRC patients,^70–73^ which underlines the importance of motivating and helping patients to cope with these unhealthy lifestyle habits. The mechanisms are not fully understood but may include increased rates of surgical complications, decreased response to radiotherapy and chemotherapy and nicotine-induced suppression of apoptosis of cancer cells and enhanced cell migration.^74–79^ Conversely, there is increasing evidence that physical activity might have a favourable influence on cancer outcomes, including common cancer symptoms such as fatigue, as well as quality of life and survival.^80–82^ The suggested and intensively studied mechanisms include, among others, reductions of whole-body and visceral fatness, metabolic dysregulation, chronic inflammation and oxidative stress as well as enhanced immune function.^83^ The results from RCTs investigating various types of physical activity and their specific short and long-term effects during and after the post-surgery period will be crucial to advance this field. The efficacy of some physical activity-based interventions on such outcomes has already been demonstrated by several RCTs, suggesting that treatment and surveillance of the cancer itself should be routinely supplemented by efforts to promote physical activities tailored to the individual patient’s specific conditions, in both short and long timeframes.

Important exceptions among CRC risk factors that influence survival might be overweightness and obesity. Although these factors are positively associated with CRC risk, patients who are overweight around the time of or after a CRC diagnosis seem to have lower mortality, and mortality of obese patients seems to be similar or only slightly increased compared to normal-weight patients.^84,85^ Although the underlying mechanisms are not fully understood and might partly include secondary weight loss due to advanced disease, these results suggest that tertiary prevention efforts among overweight CRC patients should not include weight control to achieve ‘normal weight’. Finally, there is currently no convincing evidence that changes in dietary factors known to be related to CRC risk, such as consumption of high levels of red and processed meat, would be related to enhanced survival of CRC patients.^86,87^ Nevertheless, having a healthy body weight, being physically active and eating a diet rich in vegetables, fruits and whole grains after diagnosis was associated with a longer survival of stage-III colon cancer patients in a chemotherapy trial in the United States, suggesting that a lifestyle consistent with the American Cancer Society guidelines may enhance prognosis.^88^

### Chemoprevention as a tertiary prevention strategy

A potential role for chemoprevention in tertiary prevention is currently subject to intensive research. There is increasing evidence from observational studies that use of low-dose aspirin, which is associated with reduced CRC risk, also goes along with enhanced survival after CRC diagnosis.^89^ The likely key mechanisms are related to COX inhibition,^90^ but an additional role of non-COX mechanisms has also been postulated. Several RCTs have been initiated to explore and help to define a potential role for aspirin in tertiary prevention.^91^ Likewise, observations of enhanced survival of CRC patients treated with metformin should be followed up by RCTs.^92^ For other drugs commonly used for treatment of comorbidities among CRC patients, such as beta-blockers, an apparent beneficial role for prognosis seems to have been spurious, and is thought to have resulted from major flaws in pertinent pharmacoepidemiological studies, such as immortal time bias.^93^

Epidemiological studies have also shown that vitamin D deficiency, which is common among CRC patients,^94^ is associated with strongly reduced chances of survival.^95,96^ The suggested mechanisms are manifold and include immunomodulatory, antiangiogenetic and proapoptotic effects of vitamin D. A recent randomised phase-II trial showed that high-dose vitamin D supplementation improved progression-free survival in metastatic colorectal cancer.^97^ A potential role for vitamin D supplementation in tertiary prevention should be further explored and corroborated by well-designed RCTs.^98^

## Summary and conclusions

Population growth, demographic ageing and unfavourable trends in major risk factors such as physical inactivity, overweightness and obesity and Western dietary habits, are likely to lead to increasing CRC incidence. Increasing numbers of cases and deaths are to be expected in the decades to come, unless effective prevention efforts are implemented. Primary prevention requires a lifetime perspective and might only pay off in the long run. Yet, given CRC shares many risk and protective factors with other common chronic diseases, including several other common cancers and various cardiovascular and metabolic diseases, primary prevention efforts aimed at reducing CRC risk factors could have benefits that extend far beyond CRC prevention and these should take high priority in cancer control.

Additional major reductions in the CRC burden are possible through effective secondary prevention. Multiple effective and cost-effective screening tools are available, including faecal immunochemical testing, flexible sigmoidoscopy and colonoscopy. Providing these screening options in the context of organised screening programmes that ensure both high adherence rates and high quality of screening offers has the potential to substantially reduce CRC mortality, even within the next 10–20 years. Screening for CRC can be highly cost-effective, if not cost-saving, and effectiveness and cost-effectiveness might further be enhanced by more risk-adapted screening strategies in the future. Furthermore, the portfolio of non-invasive or minimally invasive screening tests is expected to be expanded in the years to come by the discovery of novel molecular markers—in particular, blood-based ‘omics signatures’. Emerging evidence also suggests a potential for tertiary prevention of CRC. In particular, the use of aspirin, cessation of smoking and increasing physical activity among CRC patients have a substantially underused potential to enhance both the survival and the quality of life of CRC patients.
